# *miR-153* Regulates SNAP-25, Synaptic Transmission, and Neuronal Development

**DOI:** 10.1371/journal.pone.0057080

**Published:** 2013-02-25

**Authors:** Chunyao Wei, Elizabeth J. Thatcher, Abigail F. Olena, Diana J. Cha, Ana L. Perdigoto, Andrew F. Marshall, Bruce D. Carter, Kendal Broadie, James G. Patton

**Affiliations:** 1 Department of Biological Sciences, Vanderbilt University and Medical School, Nashville, Tennessee, United States of America; 2 Department of Biochemistry, Vanderbilt University and Medical School, Nashville, Tennessee, United States of America; Wake Forest University, United States of America

## Abstract

SNAP-25 is a core component of the trimeric SNARE complex mediating vesicle exocytosis during membrane addition for neuronal growth, neuropeptide/growth factor secretion, and neurotransmitter release during synaptic transmission. Here, we report a novel microRNA mechanism of SNAP-25 regulation controlling motor neuron development, neurosecretion, synaptic activity, and movement in zebrafish. Loss of *miR-153* causes overexpression of SNAP-25 and consequent hyperactive movement in early zebrafish embryos. Conversely, overexpression of *miR-153* causes SNAP-25 down regulation resulting in near complete paralysis, mimicking the effects of treatment with Botulinum neurotoxin. *miR-153*-dependent changes in synaptic activity at the neuromuscular junction are consistent with the observed movement defects. Underlying the movement defects, perturbation of *miR-153* function causes dramatic developmental changes in motor neuron patterning and branching. Together, our results indicate that precise control of SNAP-25 expression by *miR-153* is critically important for proper neuronal patterning as well as neurotransmission.

## Introduction

Trimeric soluble N-ethylmaleimide-sensitive factor attachment protein receptor (SNARE) complexes form the core machinery mediating vesicular exocytosis [1]–[3]. In the nervous system, SNARE complexes are involved in membrane addition during neuronal growth as well as both dense core vesicle (DCV) release of proteins and synaptic vesicle (SV) release of fast neurotransmitters. At synapses, the core SNARE protein SNAP-25 interacts with accessory proteins that together regulate SV exocytosis by linking Ca^2+^ sensing to membrane fusion and neurotransmitter release [4]–[7]. SNAP-25 is a specific target of Botulinum neurotoxin proteases that block vesicle release, resulting in rapid paralysis and death [8], [9]. Misregulation of SNAP-25 is associated with several human diseases and neurodegenerative disorders including Huntington’s Disease [10], Alzheimer’s Disease [11], and diabetes [12].

SNAP-25 is required for action potential-evoked glutamatergic, cholinergic, and glycinergic transmission in neurons [13], [14]. Mouse knockouts of SNAP-25 are therefore lethal although neuronal cultures from SNAP-25 null mutants maintain the ability to exhibit stimulus-independent transmitter release [13], [15]. GABAergic inhibitory synapses express lower levels of SNAP-25 and may be more sensitive to calcium regulation, whereas glutamatergic excitatory synapses express higher amounts of SNAP-25 that alters calcium sensitivity [4]. Part of this differential regulation could be due to accessory proteins that control SNAP-25 distribution and levels to modulate synaptic activity [16]–[18]. Transcriptional mechanisms regulating SNAP-25 levels have also been suggested to play key roles in the dynamic control of synaptic function [19]–[23].

Several miRNAs have been shown to regulate synapse formation or homeostasis, mostly within the post-synaptic dendrite [22], [24], [25]. On the presynaptic side, most forms of regulation center on modulation of calcium channels and calcium-dependent vesicle release [26], [27]. In this study, we show that *miR-153* inhibits SNAP-25 expression in the developing nervous system. Precise control of SNAP-25 by *miR-153* is necessary not only for presynaptic vesicle release, but also for protein secretion, motor neuron patterning, and outgrowth.

## Results

### *miR-153* Regulates Embryonic Movement

*miR-153* has been proposed to be one of a limited number of ancient miRNAs that evolved with the establishment of tissue identity [28]. It is conserved among bilaterians displaying distinct expression patterns in neurosecretory brain cells of the deuterostome marine worm *Platynereis dumerilii* and the protostome annelid *Capitella* [28]. In zebrafish, *miR-153* is expressed in distinct regions of the developing nervous system and brain, including neurosecretory cells of the hypothalamus [29], [30]. Using deep sequencing and *in situ* localization, we detected robust *miR-153* expression in the developing zebrafish brain and reduced, but detectable levels in the spinal cord as early as the 18 somite stage, with progressively increasing expression thereafter [30], [31] [32].

**Figure 1:**
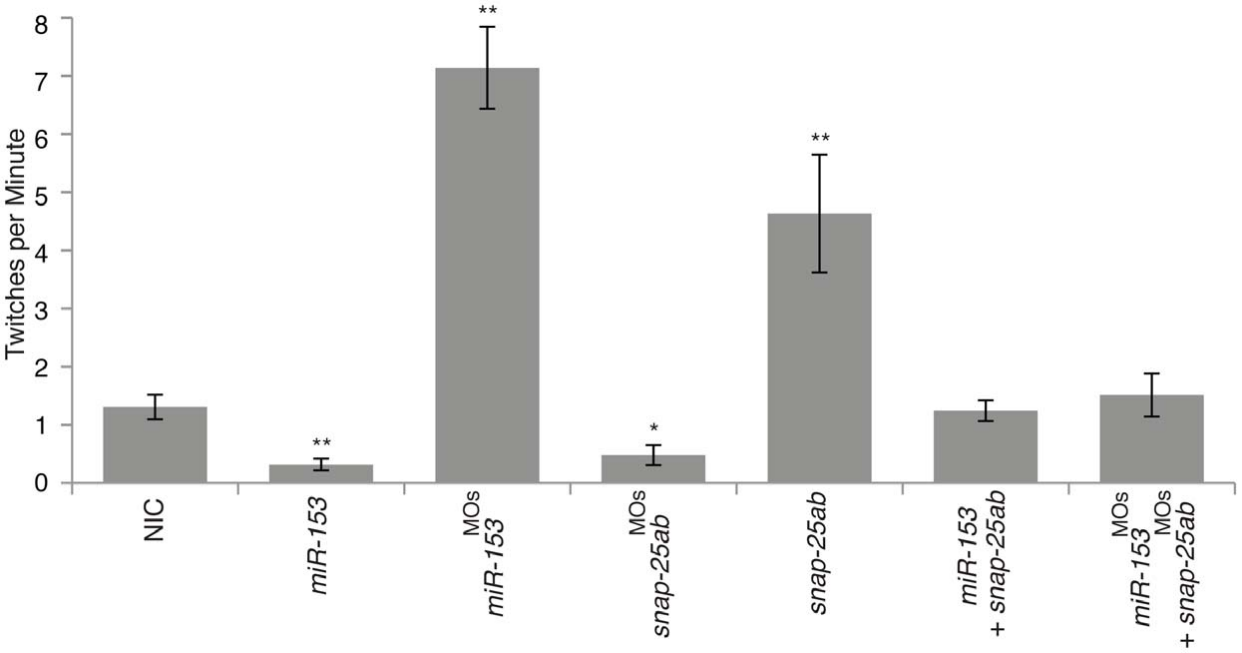
*miR-153* regulates embryonic movement. Embryonic movement was recorded at 1 dpf for each of the singly and multiple injected conditions shown (see Movies). The number of twitches per minute was counted and significance determined by comparing the noninjected control (NIC) embryos to all other conditions using ANOVA with Dunnett’s post-test. *, p<0.05; **, p<0.01. Movements were counted for approximately 60 embryos over 2–5 minutes for each condition.

To determine the function of *miR-153*, we injected either synthetic *miR-153* or antisense morpholinos against *miR-153* into single cell embryos and allowed development to proceed for 1–2 days. Two different morpholinos were used to ensure specificity and we verified overexpression and knockdown of *miR-153* using northern blots (Fig. S1). No gross morphological changes were observed in injected embryos and normal localization of neuronal markers was detected at the midbrain-hindbrain boundary, inner ear, and retina at 1–2 dpf (data not shown). Despite the lack of morphological changes, we observed striking behavioral movement defects in injected embryos. To quantify movement, embryos were recorded over time (Movie S1) with analyses restricted to embryos within the chorion at 24 hpf. Normal zebrafish embryos move within the chorion with a characteristic frequency of ∼1 twitch/minute at 24 hpf (Fig. 1). Strikingly, embryos injected with *miR-153* were almost completely motionless, with little or no spontaneous movement, although their hearts were beating normally and minimal movement could be elicited by touch stimulation (Fig. 1). In contrast, knockdown of *miR-153* caused a dramatic and significant 7-fold increase in the frequency of spontaneous movement (Fig. 1). Interestingly, upon touch stimulation, *miR-153* morphants would initially respond with unusually robust, hyperactive movements after which all motion would cease altogether for a period of time (whether touched or not), followed by a resumption of hyperactive movement upon stimulation. At 52 hpf, *miR-153* overexpression fish embryos were still mostly motionless, while *miR-153* knockdown embryos were still hyperactive (data not shown).

### *miR-153* Targets *snap-25*

**Figure 2:**
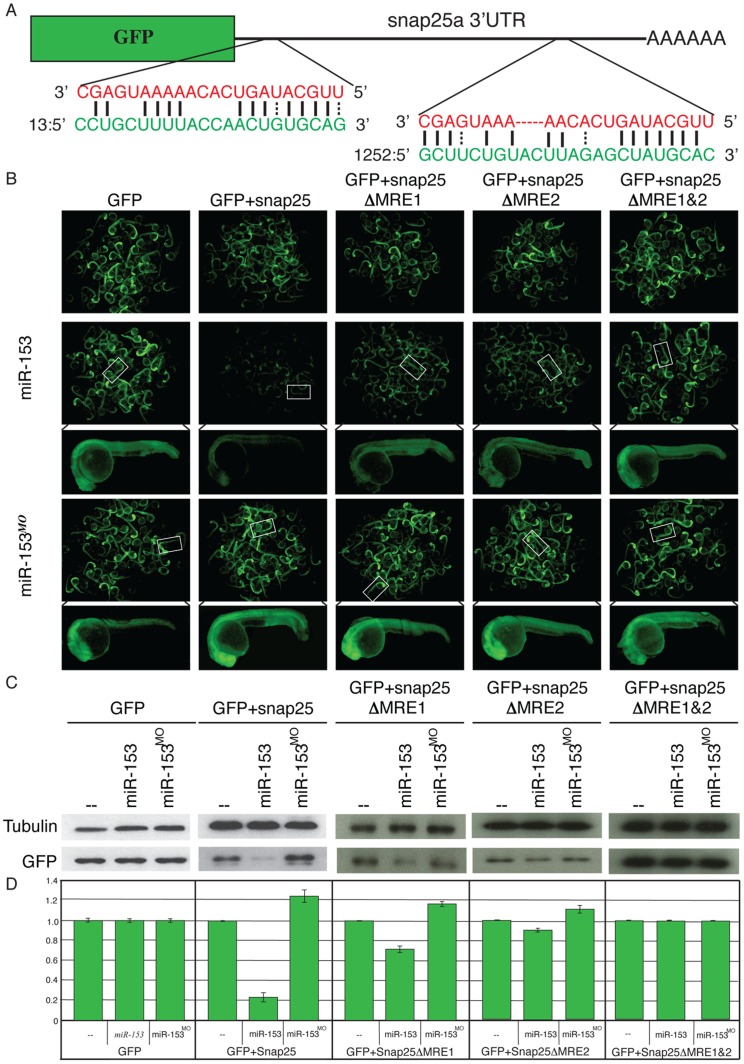
*miR-153* targets *snap-25a*. (A) GFP reporter constructs were created by fusing the reading frame of GFP to the *snap-25a* 3′UTR. Two predicted miRNA recognition elements (MREs) were identified in the *snap-25a* 3′ UTR. The *miR-153* sequence is indicated in red and the corresponding *snap-25a* UTR sequence is shown in green. (B) Single cell zebrafish embryos were injected with mRNAs derived from GFP reporters lacking a UTR (GFP), fused to the full length *snap-25a* UTR (*+snap-25),* or mutant versions of the *snap-25a* UTR lacking individual MREs (*snap-25a*ΔMRE1 and *snap-25a*ΔMRE2) or both MREs (*snap-25a*ΔMRE1&2). Embryos were injected in the presence or absence of exogenous *miR-153* or morpholinos against *miR-153* (*miR-153^MO^*). Fluorescence levels were examined at 1 dpf. Clusters of embryos (∼60) are shown as well as a high magnification image of a single representative embryo. (C) Lysates from ∼100 embryos were prepared from embryos treated as in B and GFP protein levels were determined by western blotting using antibodies against GFP or control antibodies against α-tubulin. (D) Quantitation of westerns was performed with a paired Student’s t-test (n = 5).

To identify mRNAs regulated by *miR-153*, we used target prediction algorithms, compared the expression patterns of both potential mRNA targets and *miR-153,* and assayed phenotypes from gain and loss of function experiments. Based on these criteria, *snap-25* proved to be a *bona fide* target for *miR-153* based on the results of reporter silencing experiments (Fig. 2) and consistent with conservation of miRNA recognition elements (MREs) from fish to humans (Fig. S2).

There are two SNAP-25 paralogs in zebrafish (*a* and *b* isoforms) with similar, but not identical, 3′ UTRs [33], [34]. For reporter assays, we fused the 3′ UTR from both *snap-25* isoforms to the GFP reading frame (*snap-25a* data shown in Fig. 2A; *snap-25b* shown in Fig. S3). Synthetic mRNAs prepared from these reporters were injected into single cell embryos in the presence or absence of exogenous *miR-153* or *miR-153* morpholinos (MOs). Based on fluorescence levels in live embryos at 1 dpf, co-injection of *miR-153* resulted in obvious down-regulation of GFP for both isoforms (Fig. 2B). To confirm that the loss of GFP was due to pairing with the predicted MREs, we created deletions of individual and combinations of MREs in *snap-25a* and *snap-25b*. Deletion of both MREs from *snap-25a* and all three MREs from *snap-25b* abolished the ability of *miR-153* to silence expression (Fig. 2B; Fig. S3B). For *snap-25a*, we tested each of the individual MREs and found that deletion of a single MRE resulted in only modest silencing whereas deletion of both MREs caused a loss of silencing. We conclude that *miR-153* targets both isoforms of *snap-25* in an MRE-dependent manner.

If *miR-153* targets *snap-25*, knockdown of endogenous *miR-153* should lead to increased reporter fluorescence. To test this prediction, antisense morpholinos were co-injected with reporter mRNAs (Fig. 2). We found that knockdown of *miR-153* caused a significant increase in GFP expression compared to embryos with wild type levels of endogenous *miR-153*. Lastly, we performed western blots using antibodies against GFP and analyzed protein levels in lysates prepared from pools of embryos treated as above (Fig. 2C,D). The levels of GFP mirrored the effects observed using fluorescence imaging in live embryos–reduced reporter expression in the presence of *miR-153* and increased reporter expression upon knockdown of *miR-153* (Fig. 2C,D). In all cases, the effects were dependent on intact MREs. Taken together, the *in vivo* reporter assays and western blots support the conclusion that *snap-25* is a target of *miR-153*.

**Figure 3:**
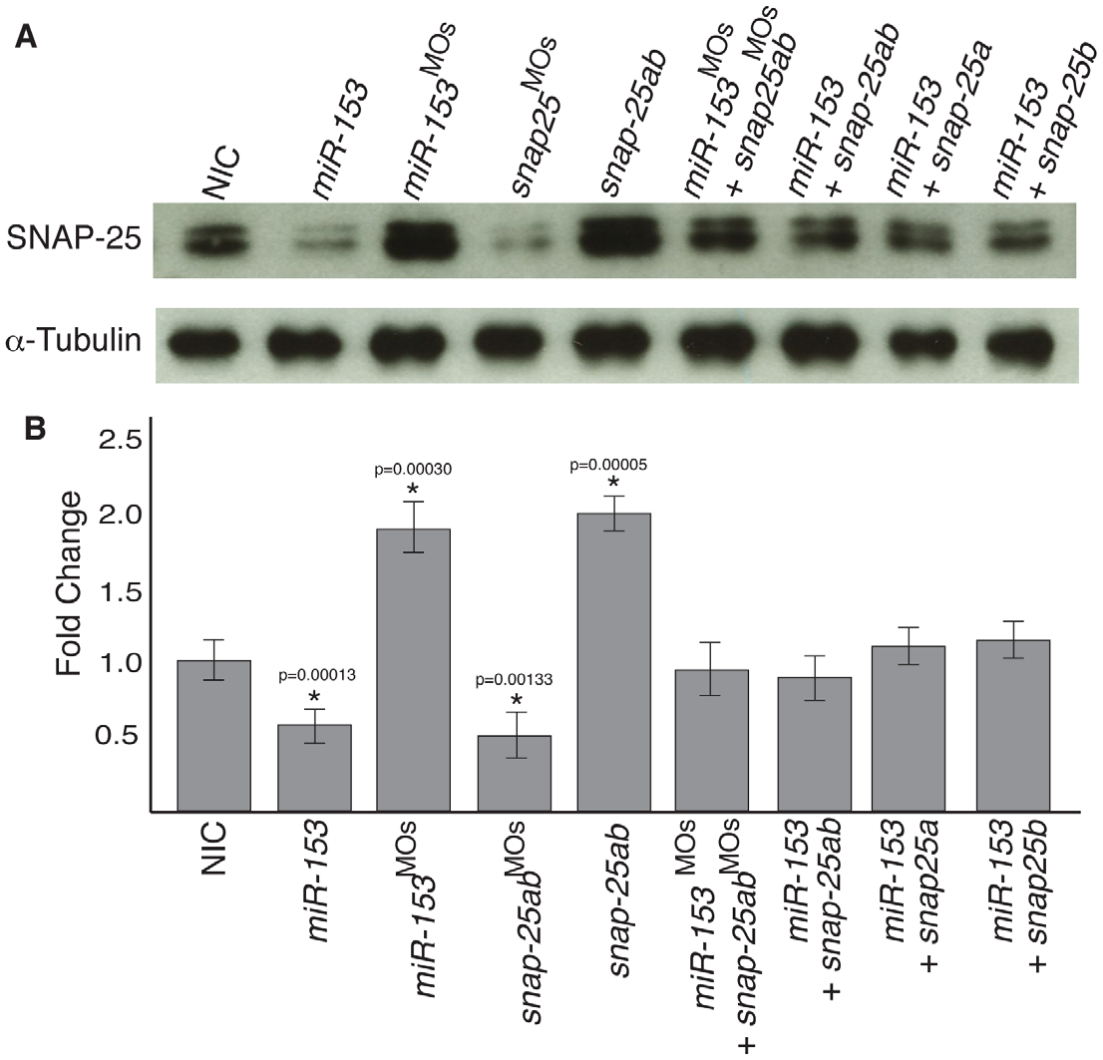
*miR-153* regulates endogenous *snap-25a* expression. (A) Embryo lysates were prepared from either NIC embryos or embryos injected with *miR-153*, *miR-153^MO^*, mRNAs encoding *snap-25a* and *snap-25b*, morpholinos against *snap-25,* or combinations thereof, as indicated. Western blots were performed using antibodies against SNAP-25 and α–tubulin. (B) Quantification of SNAP-25 levels from the western blots (n = 3) shown in A. Significance was determined by a two-tailed Student’s t-test. Error bars show s.e.m.

We next tested whether *miR-153* targets endogenous *snap-25*. Single cell embryos were injected with either *miR-153* or antisense morpholinos followed by western blots on pooled 1 dpf embryo lysates using antibodies against SNAP-25. Titration experiments were performed to optimize the levels of injected reagents (Figs. S4,S5). After optimization, protein levels were analyzed and fold changes in expression were determined compared to the amounts detected in noninjected controls (NIC) (Fig. 3). Under these conditions, excess *miR-153* led to a ∼50% decrease in SNAP-25 levels whereas knockdown of endogenous *miR-153* increased SNAP-25 levels ∼2-fold. To test for specificity we co-injected embryos with combinations of *miR-153*, *snap-25a,b* mRNAs, or morpholinos against both (Fig. 3). Injection of mRNAs encoding *snap-25a,b* resulted in a 2-fold elevation in SNAP-25 levels whereas injection of morpholinos that block the translation start site of *snap-25* led to a ∼50% decrease in SNAP-25 levels. Importantly, co-injection of combinations of RNAs and morpholinos could suppress these effects and rescue SNAP-25 levels (Fig. 3). For both suppression experiments, the effects were dose dependent. Even though *snap-25a* was more effective than *snap-25b* at rescuing endogenous SNAP-25 levels, combinations both were the most effective (Fig. 3). These results indicate specific targeting of *snap-25* by *miR-153*. Although *miR-153* is likely to have additional targets, the ability to specifically rescue the effects of overexpression and knockdown of both *miR-153* and *snap-25* indicates that the effects we observe are specific to targeting of *snap-25* by *miR-153*.

### *miR-153* Regulates *snap-25* to Control Movement

Because we could specifically suppress the effects of overexpression or knockdown of *miR-153* by co-injection of either *snap-25a,b* mRNA or morpholinos against *snap-25a,b*, we next sought to test whether the movement defects are caused by altered *miR-153* levels could likewise be rescued in a *snap-25* dependent manner. Embryonic movements were quantitated at 24 hpf after injection of antisense morpholinos against *snap-25* (*snap25^MO^*) or with *snap-25a,b* mRNAs (Fig. 1; Movie S1). Knockdown of *snap-25* resulted in dramatically decreased embryonic movements, similar to overexpression of *miR-153* (Fig. 1). In contrast, overexpression of *snap-25a,b* increased movement approximately 5-fold over control NIC embryos (Fig. 1). For rescue experiments, co-injection of *snap-25a,b* mRNA with *miR-153* restored near normal movement (Fig. 1; Movie S1). Similarly, co-injection of morpholinos against both *snap-25* and *miR-153* also restored normal movement (Fig. 1; Movie S1). Thus, not only were SNAP-25 protein levels restored to normal, but also movement defects were rescued, demonstrating specific targeting of *snap-25* by *miR-153*.

**Figure 4:**
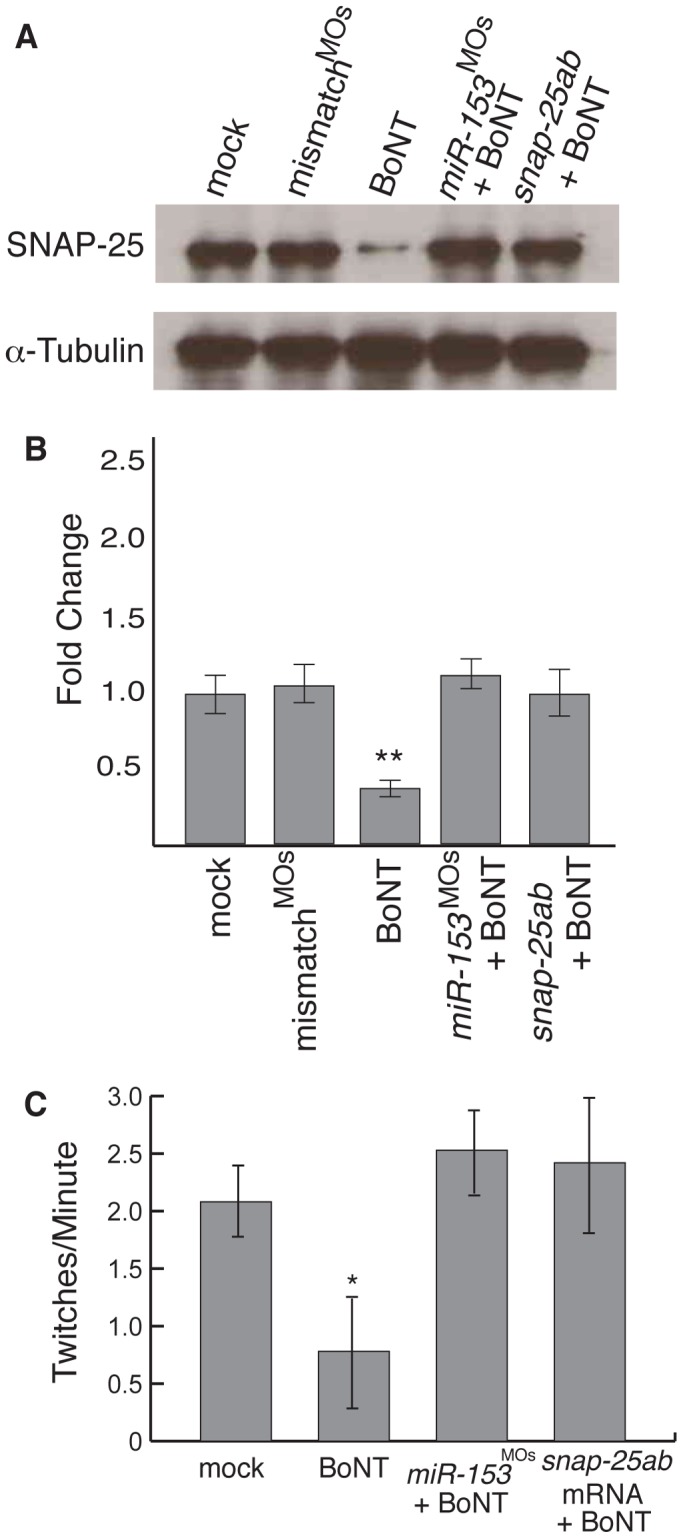
*miR-153* mimics the effects of BoNT A. (A) Single cell embryos were injected as indicated and then at 27 hpf, exposed to Botulinum neurotoxin A (BoNT) for 30 minutes. After recovery for 1 hour, western blots were performed on embryo lysates using antibodies against SNAP-25 or α–tubulin. (B) Quantitation of SNAP-25 levels from A, n = 3. **, p<0.01 (C) Embryonic movement in the presence or absence of BoNT A. The number of twitches per minute was counted as in Fig. 1 for embryos treated as indicated. Significance was determined by comparing mock embryos to all other conditions using ANOVA with Dunnett’s post-test, n = 15. *, p<0.05.

SNAP-25 is a known target of Botulinum neurotoxin (BoNT) proteases A and E [8], [9]. If *miR-153* is targeting *snap-25*, the effects of increased *miR-153* should mimic the effects of BoNT A. To test this prediction, injected zebrafish were exposed to BoNT A for 30 minutes at 27 hpf. One hour later, western blots were performed on pooled protein samples to determine whether it was possible to rescue SNAP-25 over-expression phenotypes associated with *miR-153* knockdown or injection of *snap25a,b* mRNAs. Exposure to BoNT A dramatically reduced SNAP-25 levels, recapitulating the effects of *miR-153* knockdown and over-expression (Fig. 4A,B). For movement, exposure to BoNT A rescued the hyperactive phenotypes observed after injection with MOs against *miR-153* or overexpression of *snap-25a&b* mRNAs (Fig. 4C; Movie S1). Together, these experiments strongly support the conclusion that *miR-153* specifically targets *snap-25* to regulate embryonic movement.

### *miR-153* Regulation of Motor Neuron Development

SNAP-25 is a well-characterized t-SNARE protein, with an established function in vesicular exocytosis [1]–[3]. In the developing nervous system, the SNARE complex mediates vesicular membrane addition driving neurite outgrowth and morphological patterning [1]–[3], [35]. Moreover, DCV-mediated release of signaling proteins and growth factors is important for axon guidance, path finding, and morphological development [36]–[39]. We therefore sought to determine whether *snap-25* regulation by *miR-153* would alter neuronal morphogenesis. Because zebrafish motor neuron development is well characterized [40]–[45], we decided to focus on the effects of *miR-153* on motor neurons during early zebrafish development.

**Figure 5:**
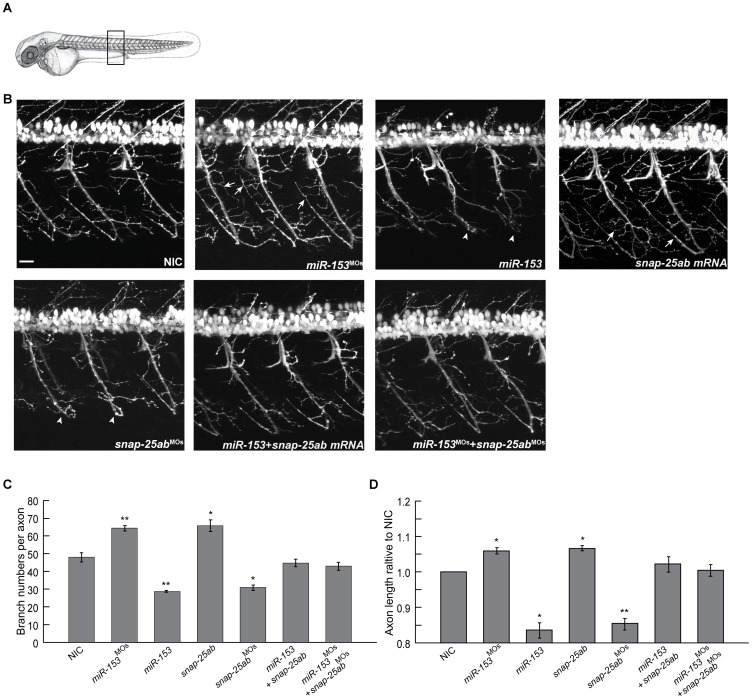
*miR-153* regulates the morphology and structure of motor neurons. (A) A transgenic zebrafish line, *Tg(mnx1:TagRFP-T)*, that expresses RFP in motor neurons was used to monitor the effects of altered levels of *miR-153* and *snap-25* at 55 hpf. For all confocal images, developing motor neurons were examined from the same somites, as indicated. (B) Morphology of developing motor neurons under each of the indicated conditions. Arrows indicate increased branching after knockdown *miR-153* (*miR-153^MO^*) or overexpression *snap-25a,b* mRNA. Arrowheads indicate the structural defects after *miR-153* overexpression or knockdown of *snap-25a,b* (*snap-25a,b^MO^*). Scale bar: 20 µm. (C) Quantification of motor neuron axonal branch number under the different conditions shown in (B). Error bars show s.e.m. Significance was determined using ANOVA with Dunnett’s post-test, n = 5. *, p<0.01; **, p<0.005. (D) Quantification of motor neuron axon length relative to uninjected control under the different conditions shown in (B). Error bars show s.e.m. ANOVA with Dunnett’s post-test, n = 5. *, p<0.05; **, p<0.01.

We first injected *miR-153* or morpholinos against *miR-153* to observe the effects on the development and morphology of motor neurons in a transgenic zebrafish line in which motor neurons are specifically labeled with RFP (*Tg(mnx1:TagRFP-T*) [46]. Perturbation of *miR-153* levels caused striking changes in motor neuron structure and branching (Fig. 5A,B). Compared with NICs, overexpression of *miR-153* dramatically changed the axonal architecture with significant decreases in branch numbers and length (Fig. 5C, D). Knockdown of *miR-153* resulted in completely opposite effects with increased motor projection architectural complexity, increased axonal length, and increased branch numbers (Fig. 5B–D). To test whether the effects were specific, we conducted rescue experiments, as above. Injection of *snap-25a,b* mRNA or morpholinos against *snap-25a/b* produced virtually the same phenotypes observed in embryos subjected to *miR-153* knockdown or overexpression, respectively. In contrast, co-injection of *miR-153* and *snap-25a,b* mRNAs or morpholinos against *miR-153* and *snap-25a,b* almost completely restored the normal patterning and branching of motor neurons (Fig. 5B–D). These results indicate that *miR-153* regulates motor neuron development via control of *snap-25a,b*.

**Figure 6:**
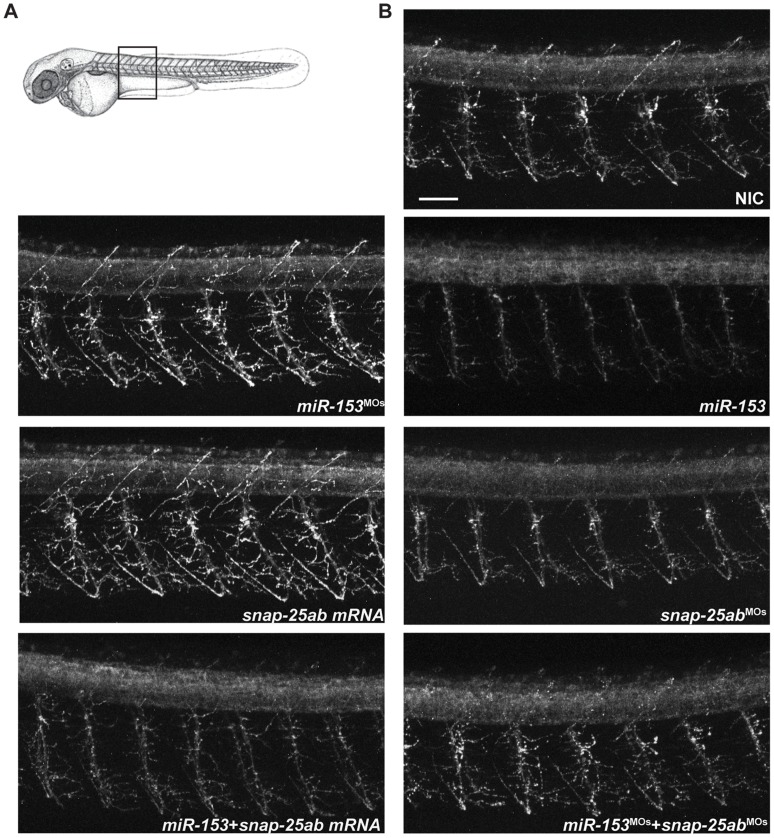
*miR-153* regulates primary motor neuron development. (A) Immunofluorescence performed on whole mount zebrafish embryos at 55 hpf using Znp-1 antibodies to label primary motor neurons. Confocal images were acquired from the same somites for all embryos, as indicated. (B) Effects on primary motor neuron structure and branching under the indicated conditions. Scale bar: 40 µm.

To further dissect the function of *miR-153* on motor neuron development, immunofluorescence was performed on whole-mount zebrafish embryos (55 hpf) with antibodies that label primary (Znp-1 or anti-synaptotagmin 2) or secondary (Zn-8 or Alcama) motor neurons [47]. Compared to NIC embryos, a striking difference in primary motor neuron axon architecture was observed with both *miR-153* overexpression (*miR-153*) and knockdown (*miR-153^MO^*)(Fig. 6). A significant decrease in branching was observed in *miR-153* injected embryos whereas knockdown of *miR-153* caused a dramatic increase in branching. Likewise, injection of *snap-25a,b* mRNA led to increased axonal growth and branching in primary motor neurons whereas knockdown of *snap-25a,b* caused decreased outgrowth and branching (Fig. 6). Co-injection experiments showed that *snap-25a,b* mRNA and morpholinos against *snap-25* could partially counteract the effects of the corresponding gain and loss of *miR-153*.

For secondary motor neurons, rostral axon outgrowth was similarly stunted and/or irregularly spaced by *miR-153* overexpression and slightly elongated by *miR-153* knockdown (Fig. S6). Differences in the caudal region were minimal compared to earlier developing rostral neurons, possibly reflecting temporal limitations to injection experiments or perhaps increased vulnerability of rostral motor neurons to altered SNAP-25 levels. Focusing on rostral effects, injection of *snap-25a,b* mRNA phenocopied *miR-153* knockdown and injection of morpholinos against *snap-25* resulted in patterns that closely resembled *miR-153* overexpression. Co-injection of morpholinos against both *miR-153* and SNAP-25 largely restored normal secondary motor neuron patterning, although the injection of *snap-25a,b* mRNAs was not as effective at rescuing the defects that resulted from *miR-153* overexpression (Fig. S6). This may indicate a possible additional function for *miR-153* in regulating axonal growth and patterning during secondary motor neuron development.

### Expression of *miR-153* in Motor Neurons

**Figure 7:**
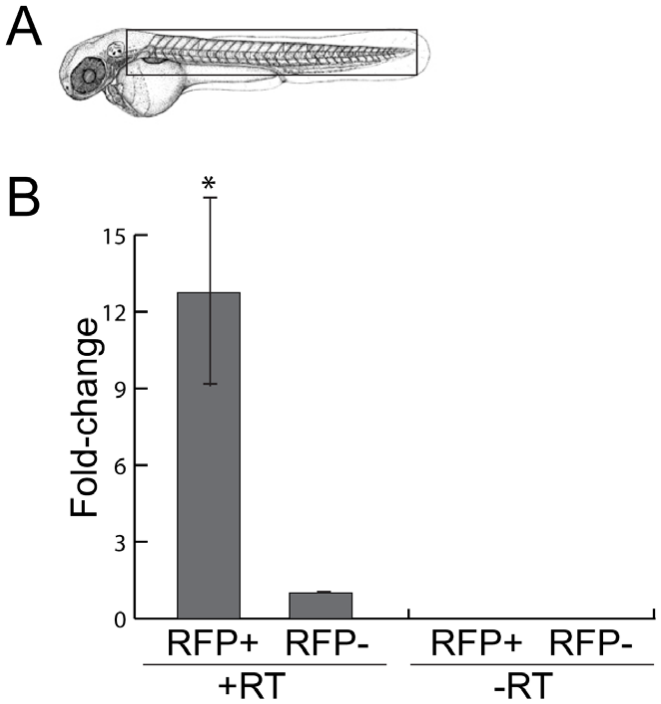
*miR-153* is expressed in motor neurons. To enrich for motor neurons, heads were removed from 52 hpf embryos just posterior to the otic vesicle and trunks were dissociated to facilitate sorting of RFP+ and RFP- cells. RNA was isolated from these cell fractions and RT/PCR was performed to determine *miR-153* levels relative to U6 snRNA. Significance was determined by a two-tailed Student’s t-test with the error bars representing s.e.m.; p<0.02.

To ensure that the effects of *miR-153* on motor neuron patterning were due to expression of *miR-153* in these cells, we FACS sorted cells from the trunks of 52 hpf (*Tg(mnx1:TagRFP-T*) embryos and conducted RT/qPCR. As shown in Fig. 7, there was a greater than 10-fold enrichment for *miR-153* in RFP+ cells compared to RFP- cells. Prior work had shown that *miR-153* is expressed in the brain and spinal cord but these results show that *miR-153* is expressed in developing motor neurons.

### *miR-153* Regulates Vesicular Exocytosis to Control Signaling

**Figure 8:**
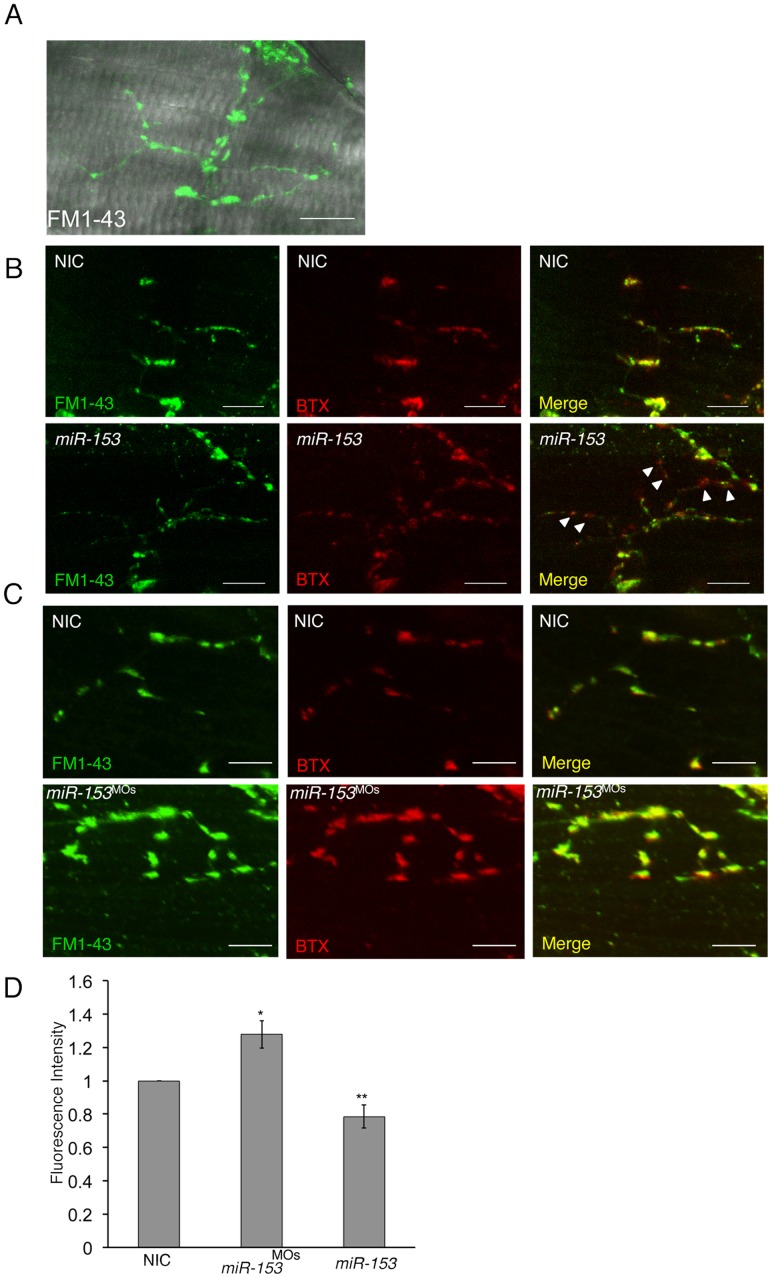
*miR-153* regulates synaptic activity at the neuromuscular junction. (A) FM1-43 loading of neuromuscular junction (NMJ) boutons in 55 hpf fish embryos. (B) Postsynaptic clusters of AChRs were labeled with Alexa 594-conjugated α-bungarotoxin. Overexpression of *miR-153* caused decreased FM1-43 loading, indicating down-regulation of the synaptic vesicle cycle within NMJ boutons (arrowheads). (C) Knockdown of *miR-153* (*miR-153^MO^*) promoted greater uptake of FM1-43 dye, indicating increased synaptic vesicle cycling. Scale bar: 10 µm. (D) Quantification of FM1-43 fluorescent intensity with a paired Student’s t-test. Error bars show s.e.m. *p<0.01; **p<0.02.

Since SNAP-25 has a well-established function in the fusion and release of numerous vesicle types, we next examined the role that *miR-153* plays in modulating exocytosis. Owing to the core role of *miR-153* in movement control, we first focused on synaptic activity at the neuromuscular junction (NMJ) in zebrafish embryos. For this analysis, we measured synaptic vesicle (SV) cycling using the styryl dye, FM1-43 [48], [49]. At 55 hpf, embryonic NMJs were imaged with Alexa 594-conjugated α-bungarotoxin (α-Btx) to label postsynaptic acetylcholine receptor (AChR) clusters, while monitoring FM1-43 uptake into NMJ presynaptic boutons (Fig. 8). The terminals were acutely depolarized for 5 minutes with high [K^+^] saline (45 mM) to drive the SV cycle and load FM1-43, whereas only weak loading was evident in low [K^+^] conditions. In non-injected controls, fluorescence was observed along terminal axon branches with intense staining at individual synaptic varicosity boutons (Fig. 8A). Compared to NIC labeling, *miR-153* overexpression resulted in a significant decrease in FM1-43 loading in presynaptic terminals, indicating slowing of the SV cycle (Fig. 8B). In sharp contrast, knockdown of *miR-153* showed a significant increase in FM1-43 loading, indicating an elevated SV cycling rate (Fig. 8C). The significant difference between *miR-153* knockdown and overexpression conditions indicates that *miR-153* plays an important role in controlling the rate of vesicle cycling (Fig. 8D). Together, these results reveal a key function for *miR-153* in the control of presynaptic vesicle release at the embryonic NMJ, consistent with a role for *miR-153* in the regulation of embryonic movement. The overall effects on movement are therefore a combination of effects on motor neuron development and patterning as well as overall exocytic activity.

**Figure 9:**
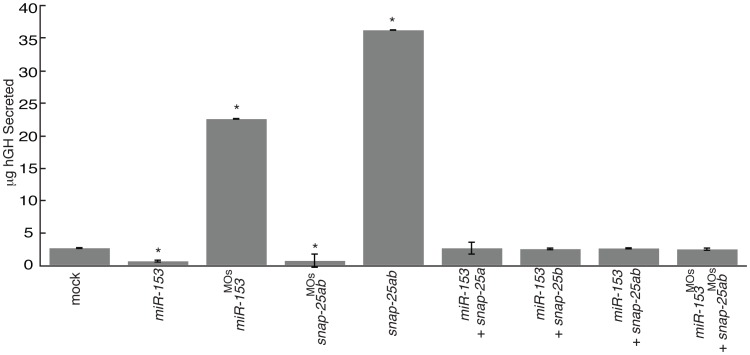
*miR-153/snap-25* regulates vesicular exocytosis. GH4C1 cells stably expressing human growth hormone (hGH) were transfected, as indicated. The effects of exogenous expression on hGH levels secreted into the culture media were determined by ELISA using hGH antibodies. Significance was determined by comparing mock transfected to all other treatments using ANOVA with Dunnett’s post-test. Error bars show s.e.m. *, p<0.01.

SNAP-25 has a highly conserved role mediating vesicular fusion in both neurons and other neurosecretory cells where it is critical for DCV release [50]. To test whether *miR-153* plays a role in this secretory context, we examined exocytosis in a rat neuroendocrine pituitary cell line (GH4C1) expressing human growth hormone (hGH) [51]. Release of hGH in these cells provided a functional readout of exocytic activity (Fig. 9). GH4C1 cells were therefore transfected with *miR-153*, morpholinos against *miR-153*/*snap-25*, or vectors expressing *snap-25a,b,* followed by determination of hGH levels in the media by ELISA. Overexpression of *miR-153* and knockdown of *snap-25a,b* (*snap-25a,b^MO^*) reduced the levels of hGH to below the amount detected in culture media from mock transfected cells (Fig. 9). In sharp contrast, knockdown of *miR-153* and overexpression of *snap-25* both significantly increased the amount of secreted hGH 8–10 fold over the mock transfected control (Fig. 9). The differences observed due to perturbation of *miR-153* levels in the GH4C1 cell line compared to embryonic NMJs are most likely due to differences in the efficiency of *miR-153*/*miR-153* ^MO^ delivery between the two experiments, as well as developmental differences. Nevertheless, the effects in this case were fully suppressed by co-expression of either *miR-153/snap-25a,b* mRNA or MOs against *miR-153/snap-25a,b*, demonstrating specific regulation of *snap-25* by *miR-153*. These data strongly support the conclusion that *miR-153* functions to precisely control SNAP-25 levels to regulate vesicle exocytosis.

## Discussion

In this study, we show that *miR-153* regulates the critical core SNARE component, SNAP-25, to modulate exocytosis and neuronal development. Increased *miR-153* levels cause decreased SNAP-25 expression resulting in decreased embryonic movement, decreased neuronal secretion, and decreased neuronal growth/branching. Conversely, *miR-153* knockdown causes elevated SNAP-25 expression resulting in hyperactive movement, increased neuronal secretion, and increased neuronal growth/branching. Accumulating evidence suggests that SNAP-25 misregulation plays a role in numerous human disease states including ADHD, schizophrenia, bipolar I disorder, Huntington’s disease, Alzheimer’s disease, and diabetes [52]. Regulated expression of *miR-153* provides an attractive model to mechanistically explain tight control of SNAP-25 levels.

### SNAP-25 Functions during Development

It is well established that axon outgrowth during neuronal development occurs via SNARE-dependent addition of membrane for growth cone extension [35], [53]. Axonal growth, pathfinding, and target recognition are secondarily modulated by SNARE-dependent release of developmental signals via dense core vesicle (DCV) exocytosis [54]–[59]. The outgrowth of both axons and dendrites is blocked by Botulinum neurotoxins A and C1, proteases specific for SNAP-25, demonstrating a direct role of SNAP-25 in neuronal morphogenesis [55], [56], [60]. Likewise, inhibition of SNAP-25 by antisense oligonucleotides blocks axonal outgrowth [54]. In stark contrast, neuronal outgrowth was surprisingly not inhibited in SNAP-25 null mice [13]. The explanation for this inconsistency is not clear. Our results show a clear requirement for SNAP-25 in motor neuron outgrowth and branching in zebrafish. It is possible that the requirement for SNAP-25 may be species specific but we found that altered levels of *miR-153* caused similar branching defects in rat PC12 cells as observed in zebrafish motor neurons, strongly arguing against this (data not shown). Perhaps the differences are due to cell-specific requirements for SNAP-25. In the retina, for example, SNAP-25 is expressed in a dynamic spatiotemporal pattern and such differential expression may underlie specific development of cholinergic amacrine cells and photoreceptors [61]. An intriguing possibility based on the results presented here is that developmental, stage-specific and/or cell-specific expression of *miR-153* may similarly regulate SNAP-25 levels, which then drives developmental and cell-specific effects.

### SNAP-25 in Synaptic Vesicle Exocytosis

SNAP-25 is one of three SNARE proteins that contribute α-helices that mediate fusion between synaptic vesicles and presynaptic membranes [1], [3]. Blockage of synaptic transmission by Clostridium and Botulinum neurotoxins first established that SNARE proteins are critical for neurotransmitter release [62]. Cleavage of SNAP-25 by Botulinum neurotoxin A causes a paralytic phenotype that resembles the loss of movement we observe in zebrafish embryos expressing excess *miR-153*. SNAP-25 haploinsufficient mice show no observable phenotypic defects but complete loss of SNAP-25 blocks evoked synaptic transmission [13]. Moreover, overexpression of SNAP-25 inhibits normal calcium responsiveness and can impair memory-associated synaptic plasticity [63]. These findings suggest that modulation of SNAP-25 levels are important for overall SNARE function, especially in generating differences in calcium dependence between neuronal and non-neuronal secretory vesicular fusion events. Matteoli and colleagues (2009) have shown that SNAP-25 is differentially expressed between excitatory glutamatergic and inhibitory GABAergic neurons in a developmental-specific manner [4]. These results remain controversial, as earlier studies did not observe this difference, but the data are consistent with an important role for SNAP-25 as a required component for both glutamatergic and GABAergic transmission [64], [65]. Mechanisms for how SNAP-25 levels might be regulated in a development- and/or cell-specific manner are uncertain, but our data strongly support miRNA regulation as a likely candidate and a critical mechanism controlling SNAP-25 levels. A recent report describing the effects of chronic overexpression of SNAP-25 in the rat dorsal hippocampus demonstrated the critical importance of controlling SNAP-25 levels [63]. Elevated expression of SNAP-25 produced increased levels of secreted glutamate with cognitive deficits similar to those observed in ADHD and schizophrenia. We propose that *miR-153* control of SNAP-25 levels allows for precise regulation of SNAP-25 during development and exocytosis.

### miRNAs Regulation of Neuronal Morphogenesis and Synaptic Activity

Localized translation control in synaptic dendrites is common, requiring repression of mRNA translation during transport. miRNA mediated inhibition of translation is an attractive mechanism that can precisely control gene expression in neurons. Consistent with this hypothesis, many miRNAs are neuron or brain specific [66]. Moreover, the effector complexes that carry out repression of translation (RNA Induced Silencing Complexes; RISCs) are composed of several subunits that have been implicated in both neuronal function and disease [22], [24], [67]. For example, nervous system specific miRNAs have been shown to regulate the maturation of dopamine neurons in the midbrain as well as control serotonin transport by regulating the serotonin transporter [68], [69]. Likewise, *miR-1, miR-124, miR-125b, miR-132*, *bantam*, *miR-34* and the *miR-310* cluster have all been implicated in the modulation of synaptic homeostasis [70]–[76]. Similarly, synaptic plasticity is reportedly regulated by *miR-134* through targeting of SIRT1 or Limk1, which control dendritic spine morphogenesis [77], [78]. In addition, *miR-124* in retinal ganglion cell growth cone was shown to act through CoREST to regulate the intrinsic temporal sensitivity to Sema3A, a guide cue during axonal pathfinding and morphogenesis [79]. Our work demonstrates that *miR-153* is a member of this subset of miRNAs implicated in neuronal function but by a distinctly different mechanism through targeting of *snap-25*. *miR-153* also likely targets other mRNAs [80], but SNAP-25 regulation alone is required and sufficient to explain the role of *miR-153* regulation of movement, motor neuron morphogenesis, and SNARE-mediated secretion.

## Materials and Methods

### Ethics Statement

The Animal Care and Use Committee monitors all animal care and research at Vanderbilt. Vanderbilt University has on file with the Office for Protection from Research Risks of the NIH an Assurance of Compliance with Public Health Service regulations and requirements and provisions of the Animal Welfare Act. All zebrafish experiments in this paper were approved by the Vanderbilt University Institutional Animal Care and Use Committee (IACUC) under protocol M-09-398. In accordance with that protocol, all necessary means were taken to avoid pain. For any manipulations that might induce pain, animals were anesthetized with a 0.15% solution of Tricaine (3-amino-benzoic acidethylester). The approved method for euthanizing zebrafish is incubation in ice water.

### Microinjections

Single cell zebrafish male and female embryos were injected with 200 pg of *miR-153*, 5 ng each of *miR-153^MO^* and *miR-153^loopMO^* and/or 100 pg of *in vitro*-transcribed, capped GFP reporter mRNA with or without the *snap-25a* or *b* 3′UTR. Zebrafish *snap-25a,b* 3′ UTR sequences were amplified by PCR and subcloned downstream of the GFP ORF in pCS2+ [81]. Rescue experiments used injections of 3 ng of *snap-25* ^StartMO^ and *snap-25* ^5’UTRMO^, 150 pg of *snap-25a,b* mRNA, 250 pg of *snap-25a* mRNA, or 300 pg of *snap-25b* mRNA without 3′UTRs.

Two different morpholinos against *miR-153* were utilized. One was perfectly complementary to the mature sequence; the second was complementary to a portion of the mature sequence and then extending into the precursor loop. Targeting of *snap-25a,b* mRNAs was performed using morpholinos against the region including the start codon.

### Botulinum Toxin Analysis

Embryos injected at the 1-cell stage were treated with purified Botulinum neurotoxin A (Metabiologics, Inc., Madison, WI). Initial titration experiments were performed testing a range of BoNT A concentrations with final selection of 1 ng per 10 ml of water for 30 minutes at either 24-hpf or 48-hpf. Embryos were washed 10 times in fresh water and then allowed to recover for 1 hour prior to protein extraction or video capture to monitor movement.

### qRT-PCR and Northern Blots

Total RNA extracted from both RFP+ and RFP- cells was reverse transcribed and qPCR reactions were carried out using Taqman miRNA assays (Life Technologies, NY) using the CFX96 Real-time PCR system (Bio-Rad), as previously described [32]. Northern blots were also performed as described [82], [83].

### Western Blots

Embryos were dechorionated, deyolked, and sonicated in lysis buffer as described [83]. Approximately 100 embryos were pooled and one-tenth of the resulting samples were loaded into each lane. Membranes were probed with antibodies against α-tubulin (Abcam, ab15246), GFP (Torrey Pines, TP401) or SNAP-25 (Alomone Labs). For detection, anti-rabbit or anti-mouse HRP-conjugated secondary antibodies were used, followed by visualization with ECL.

### GFP Reporter Analyses

Reporter analyses and western blots were as described [83]. To generate the *snap-25a,b* GFP reporters, the GFP ORF was fused to the 3′ UTR sequence of zebrafish *snap-25a* or *b*. *snap-25a,b* UTRs were cloned from zebrafish whole embryo RNA preparations using oligo d(T) primed reverse transcription followed by PCR amplification with gene specific primers. Images were acquired with a Leica MZFIII dissecting scope equipped with a fluorescent laser using a Qimaging camera with Qimaging software and imported into Adobe Photoshop for orientation and cropping.

### Immunofluorescence

Embryos were fixed in 4% PFA overnight at 4°C and then permeabilized in 0.5% TritonX-100 for 60 minutes followed by treatment with protease K (20 µg/ul) for 10 minutes at room temperature. Samples were washed in PBT-DMSO before blocking overnight at 4°C (PBT-DMSO, 2% BSA, 5% goat serum). Primary antibodies (SNAP-25, 1∶1000; SV-2, 1∶300; ZNP-1, 1∶2000; ZN-8, 1∶25) were incubated overnight at 4°C, washed with PBT-DMSO, and then embryos were incubated with Cy5 or Cy3-conjugated donkey anti-mouse or rabbit antibodies (Jackson Immuno) for 4 hrs at room temperature. Before mounting and visualization, embryos were washed with PBT-DMSO. PC12 cells were fixed in 4% PFA for 15 mins, washed in PBS before incubating with primary antibodies for 1 hr, washed, incubated with secondary antibodies for 1 hr, Hoechst dye for 5 mins, washed, and visualized.

### Tissue Dissociation and Motor Neuron Isolation

*Tg(mnx1:TagRFP-T)* zebrafish embryos of 52 hpf were dechorionated, deyolked, and then dissected just posterior to the otic vesicle to collect trunks (excluding the hearts). Tissues were kept in buffer (1×PBS, pH 6.4, 1%BSA) and then dissociated using 16 U/ml papain and 0.2 U/ml Dispase (Worthington, NJ) for 30 mins at 28°C on a rotator. After complete dissociation of the tissue by careful pipetting up and down, cells were pelleted at 8000×g for 2 mins. Resuspended cells were then treated with 1 mg/ml leupeptin (Worthington, NJ) and 100 U/ml DNaseI (Sigma-Aldrich) in PBS at pH 7.4 containing 2 mg/ml MgCl_2_ for 10 mins at room temperature and then kept on ice for RFP+ and RFP- cell isolation. Gating was based on cell size and fluorescence intensity, determined by the control sample of dissociated cells from WT fish at the same developmental stage.

### FM1-43 Dye Labeling

Embryos at 55 dpf were incubated in HBSS (137 mM NaCl, 5.4 mM KCl, 1 mM MgSO_4_, 0.44 mM KH_2_PO_4_, 0.25 mM Na2HPO4, 4.2 mM NaHCO3, 1.3 mMCaCl2, 5 mM Na-HEPES) containing 0.2% Tricaine and glued onto sylgard coated glass chambers before removing the skin using a glass needle. FM1-43 and α-bungarotoxin (α-Btx) labeling procedures were as previously published [49], except the preloading incubation of FM1-43 dye was omitted and the Advasep incubation period was elongated to 15 mins. For data analysis, axons with puncta labeled with α-Btx were considered as synaptic boutons. FM1-43 puncta with sizes of 0.5–2 µm were collected for analysis using Image J.

### Cell Culture and ELISA

PC12 cells (ATCC CRL-1721) were maintained using Ham’s F12K media with 15% horse serum and 5% FBS, and transfected individually or in combination with miRNAs, mRNAs, and morpholinos. Transfections were performed with 300 nM *miR-153*, biotinylated *snap-25* MOs and *miR-153* MOs and 1.5 µg of *snap-25a,b* using Lipofectamine 2000 [84]. Co-transfection of a GFP plasmid was used to determine transfection efficiencies. Efficiencies less than 50% were discarded. One day after transfection, 50 ng/ml nerve growth factor was added to media to induce differentiation. Neurite outgrowth was assayed at day 5 by immunostaining with antibodies against acetylated α-tubulin. Stably transfected GH4C1 cells were a gift from Dr. K. Kannenberg [51]. ELISAs were performed after 5 days of transfection and human growth hormone was assayed following the Diagnostic Systems ELISA kit.

## Supporting Information

Figure S1 **Northern blot of** ***miR-153*** **overexpression and knockdown.** Perturbation of *miR-153* expression levels by injection of *miR-153* or MOs against different regions of pre-*miR-153* was verified by northern blot. U6 served as a loading control.
(TIF)

Figure S2 **Conservation of** ***snap-25*** **3′ UTR sequences.** The 3′ UTRs from mouse, human and zebrafish *snap-25a* (A) and *snap-25b* (B) are shown with the MREs that pair with *miR-153* boxed in green. Conserved nucleotides are marked by an asterisk. The exact pairings between the MREs and *miR-153* are shown in Figure 2 and Figure S3. Despite different levels of conservation, both MREs in *snap-25a* pair extensively with *miR-153* in the seed region.
(TIF)

Figure S3 ***miR-153*** **targets** ***snap-25b*** **.** (A) GFP reporter constructs were created by fusing the reading frame of GFP to the *snap-25b* 3′UTR. Three predicted miRNA recognition elements (MREs) were identified in the *snap-25b* 3′ UTR. The *miR-153* sequence is indicated in red and the corresponding *snap-25a* UTR sequence is shown in green. (B) Single cell zebrafish embryos were injected with mRNAs derived from GFP reporters lacking a UTR (GFP), fused to the full length *snap-25b* UTR (GFP*+snap-25b),* or mutant version of the *snap-25b* UTR lacking all MREs (GFP+*snap-25b*ΔMRE1, 2&3). Embryos were injected in the presence or absence of exogenous *miR-153* or morpholinos against *miR-153* (*miR-153^MO^*). Fluorescence levels were examined at 1 dpf. Clusters of embryos (∼30) are shown. (C)Lysates from ∼100 embryos were prepared from embryos treated as in B and GFP protein levels were determined by western blotting using antibodies against GFP or control antibodies against α-tubulin.
(TIF)

Figure S4 **Dose-dependent rescue of** ***miR-153*** **knockdown.** (A) Single cell embryos were injected with a constant level of *miR-153^MO^* and increasing amounts (increments of 2 ng) of *snap-25^MOs^*. Embryo lysates from ∼60 embryos in each group were prepared and SNAP-25 protein levels determined by western blotting. (B) Quantitation of westerns (n = 3) from A. The grey circle represents the amount of *snap- 25^MO^* (10 ng) used in co-injection rescue experiments.
(TIF)

Figure S5 **Dose-dependent rescue of** ***miR-153*** **over-expression.** (A) Single cell embryos were injected with a constant level of *miR-153* and increasing amounts (increments of 50 pg) of *snap-25a*, *snap-25b*, or *snap-25a&b* mRNA. Embryo lysates from ∼60 embryos were prepared from embryos in each treatment group and SNAP-25 protein levels were determined by western blotting. (B) Quantitation of westerns (n = 3) from A. The grey circles represent the amounts used in co-injection rescue experiments (75 pg each of *snap-25a* and b, 250 pg of *snap-25a*, and 300 pg of *snap-25b*).
(TIF)

Figure S6 ***miR-153*** **regulates secondary motor neuron development.** (A) Immunofluorescence was performed on whole mount zebrafish embryos at 55 hpf using Zn-8 antibodies to label secondary motor neurons. Confocal images were acquired from the same somites for all embryos, as indicated. (B) *miR-153* knockdown (*miR-153^MO^*) and *snap-25a,b* overexpression significantly increased the growth of secondary motor neuron axons (arrows). Overexpression of *miR-153* or knockdown of *snap-25a,b* (*snap-25a,b^MO^*) caused severe defects in axon development and architecture (asterisks). Scale bar: 40 µm.
(TIF)

Movie S1 **Embryo Movements in different conditions. 0:00–0:11. NIC Embryo Movements at 24 hpf** Noninjected control (NIC) zebrafish embryos at 24 hpf were filmed for one minute. Twitching was counted from individual embryos over multiple movies, as quantitated in Figure 1. **0:11–0:21. Effects of miR-153 Overexpression on Movement at 24 hpf** Single cell zebrafish embryos were injected with *miR-153* and filmed for one minute at 24 hpf. Twitching was counted from individual embryos over multiple movies, as quantitated in Figure 1. **0:22–0:32. Effects of Knockdown of** ***miR-153*** **on Movement at 24 hpf** Single cell zebrafish embryos were injected with *miR-153^MOs^* and filmed for one minute at 24 hpf. Twitching was counted from individual embryos over multiple movies, as quantitated in Figure 1. **0:33–0:42. Effects of Decreased SNAP-25 Expression on Movement at 24 hpf** Single cell zebrafish embryos were injected with *snap-25a,b^MO^* and filmed for one minute at 24 hpf. Twitching was counted from individual embryos over multiple movies, as quantitated in Figure 1. **0:42–0:52. Effects of Increased SNAP-25 Expression on Movement at 24 hpf** Single cell zebrafish embryos were injected with snap-25a,b mRNA and filmed for one minute at 24 hpf. Twitching was counted from individual embryos over multiple movies, as quantitated in Figure 1. **0:52–1:02. Effects of co-Injection of** ***miR-153*** **and** ***snap-25a,b*** **on Movement at 24 hpf** Single cell zebrafish embryos were co-injected with *miR-153* and *snap-25a,b* mRNA and filmed for one minute at 24 hpf. Twitching was counted from individual embryos over multiple movies, as quantitated in Figure 1. **1:02–1:12. Effects of co-Injection of** ***miR-153^MO^*** **and** ***snap-25a,b^MO^*** **on Movement at 24 hpf** Single cell zebrafish embryos were co-injected with *miR-153^MO^* and *snap-25a,b^MO^* and filmed for one minute at 24 hpf. Twitching was counted from individual embryos over multiple movies, as quantitated in Figure 1. **1:12–1:22. NIC Embryo Movements at 28 hpf** Noninjected control (NIC) zebrafish embryos at 28 hpf were filmed for one minute at the same time that the following Movies were created. Twitching was counted from individual embryos, as quantitated in Figure 4C. **1:22–1:32. Effects of Botulinum Toxin Treatment on Movement at 28 hpf** Single cell zebrafish embryos were injected with injection dye and treated with Botulinum toxin A at 27 hpf. After a 30 min treatment, embryos were washed and allowed to recuperate for 1 hour before being filmed. Twitching was counted from individual embryos, as quantitated in Figure 4C. **1:33–1:42. Effects of Botulinum Exposure and co-Injection of** ***miR-153^MO^*** **on Movement at 28 hpf** Single cell zebrafish embryos were injected with *miR-153^MOs^* and treated with Botulinum toxin A at 27 hpf. After a 30 min treatment, embryos were washed and allowed to recuperate for 1 hour before being filmed. Twitching was counted from individual embryos, as quantitated in Figure 4C. **1:42–1:52. Effects of Botulinum Exposure and co-Injection of** ***snap-25a,b*** **mRNA on Movement at 28 hpf** Single cell zebrafish embryos were injected with *snap-25a,b* mRNA and treated with Botulinum toxin A at 27 hpf. After a 30 min treatment, embryos were washed and allowed to recuperate for 1 hour before being filmed. Twitching was counted from individual embryos, as quantitated in Figure 4C.
(MOV)
