# Supplementary figures and images for: miR-153 Regulates SNAP-25, Synaptic Transmission, and Neuronal Development
Source: PLoS One. 2013 Feb 25;8(2):e57080. doi: 10.1371/journal.pone.0057080 (PMC3581580; doi:10.1371/journal.pone.0057080)

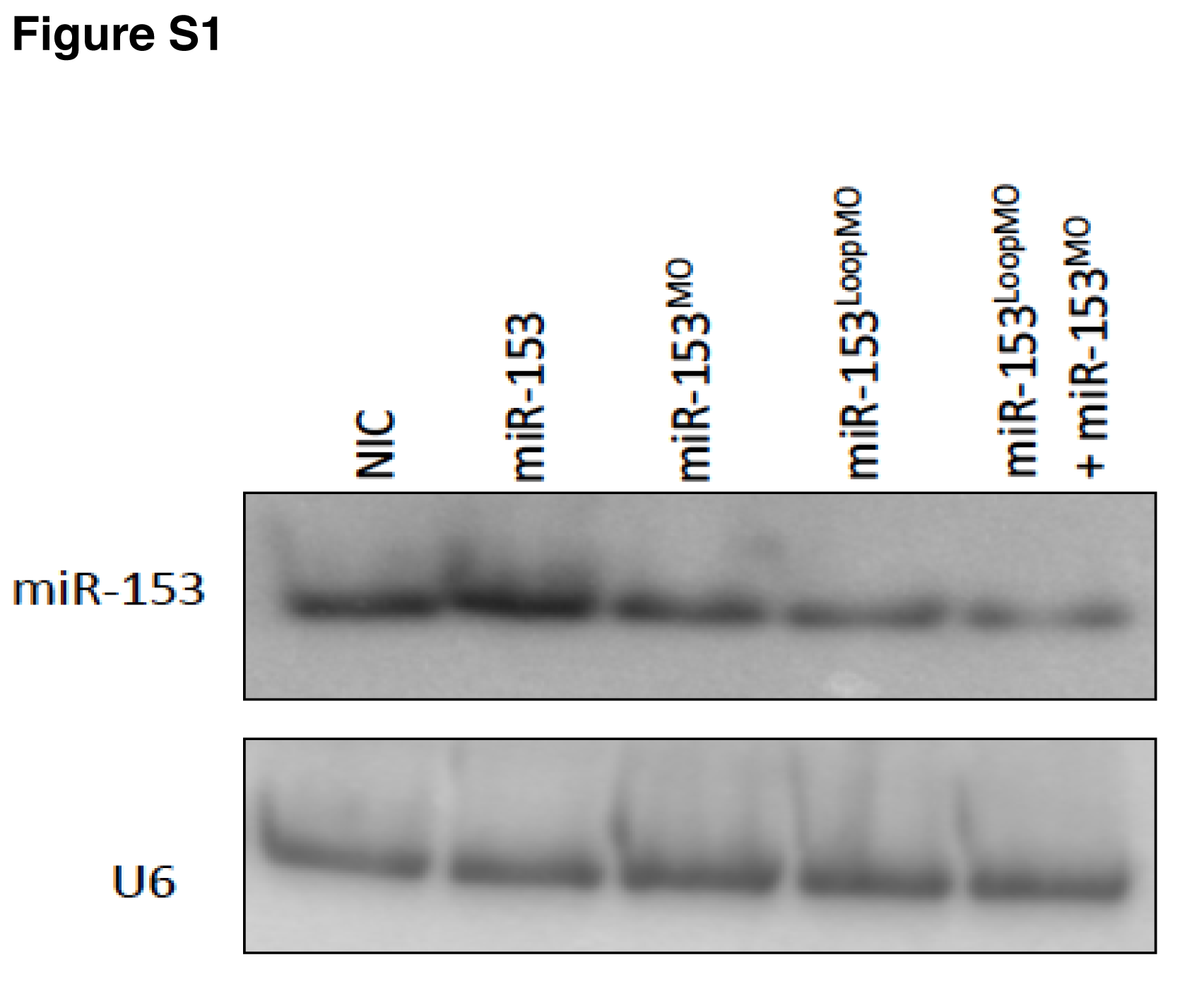

Supplement: Figure S1 — Northern blot of miR-153 overexpression and knockdown. Perturbation of miR-153 expression levels by injection of miR-153 or MOs against different regions of pre-miR-153 was verified by northern blot. U6 served as a loading control. (TIF) [file pone.0057080.s001.tif]

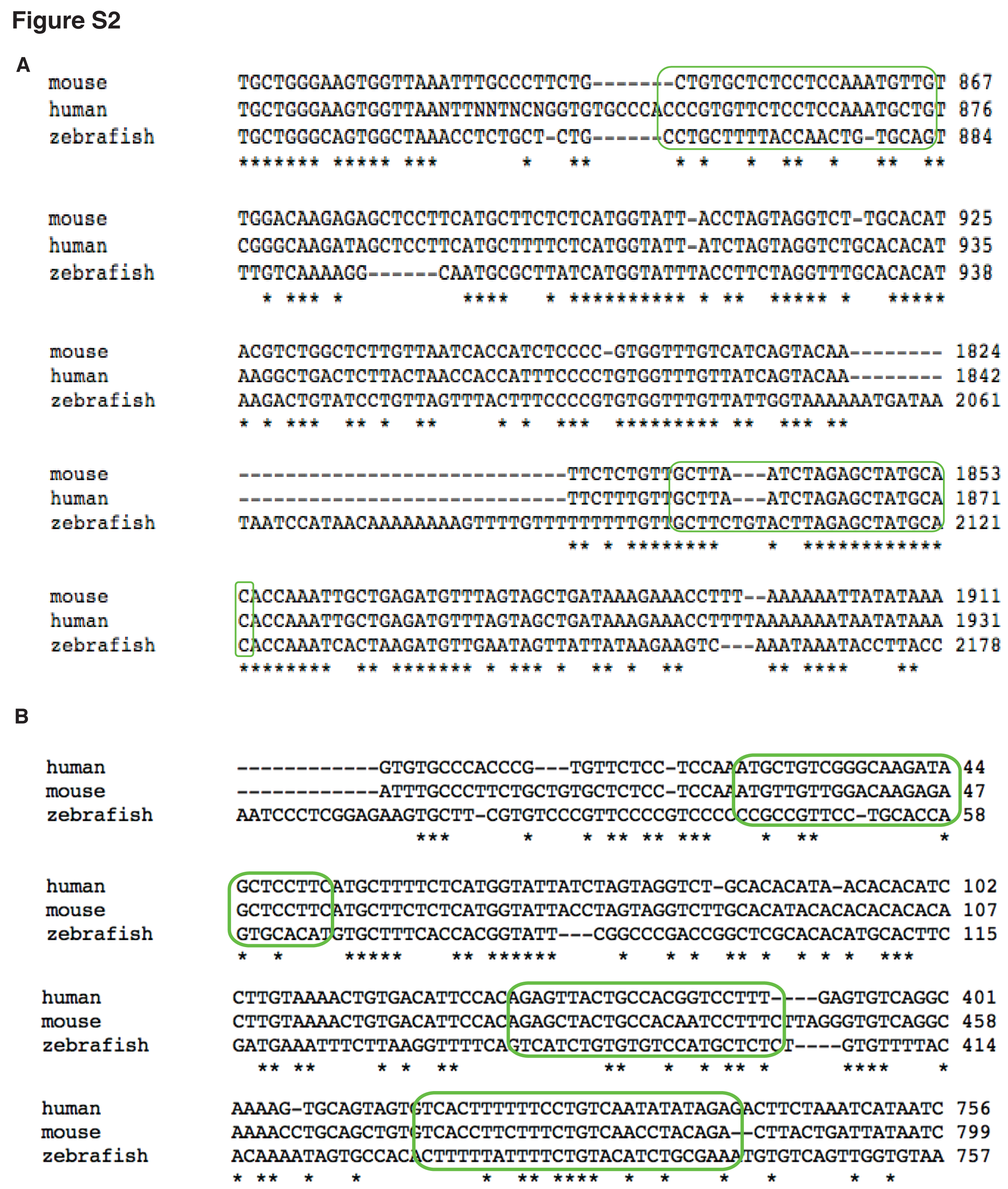

Supplement: Figure S2 — Conservation of snap-25 3′ UTR sequences. The 3′ UTRs from mouse, human and zebrafish snap-25a (A) and snap-25b (B) are shown with the MREs that pair with miR-153 boxed in green. Conserved nucleotides are marked by an asterisk. The exact pairings between the MREs and miR-153 are shown in Figure 2 and Figure S3. Despite different levels of conservation, both MREs in snap-25a pair extensively with miR-153 in the seed region. (TIF) [file pone.0057080.s002.tif]

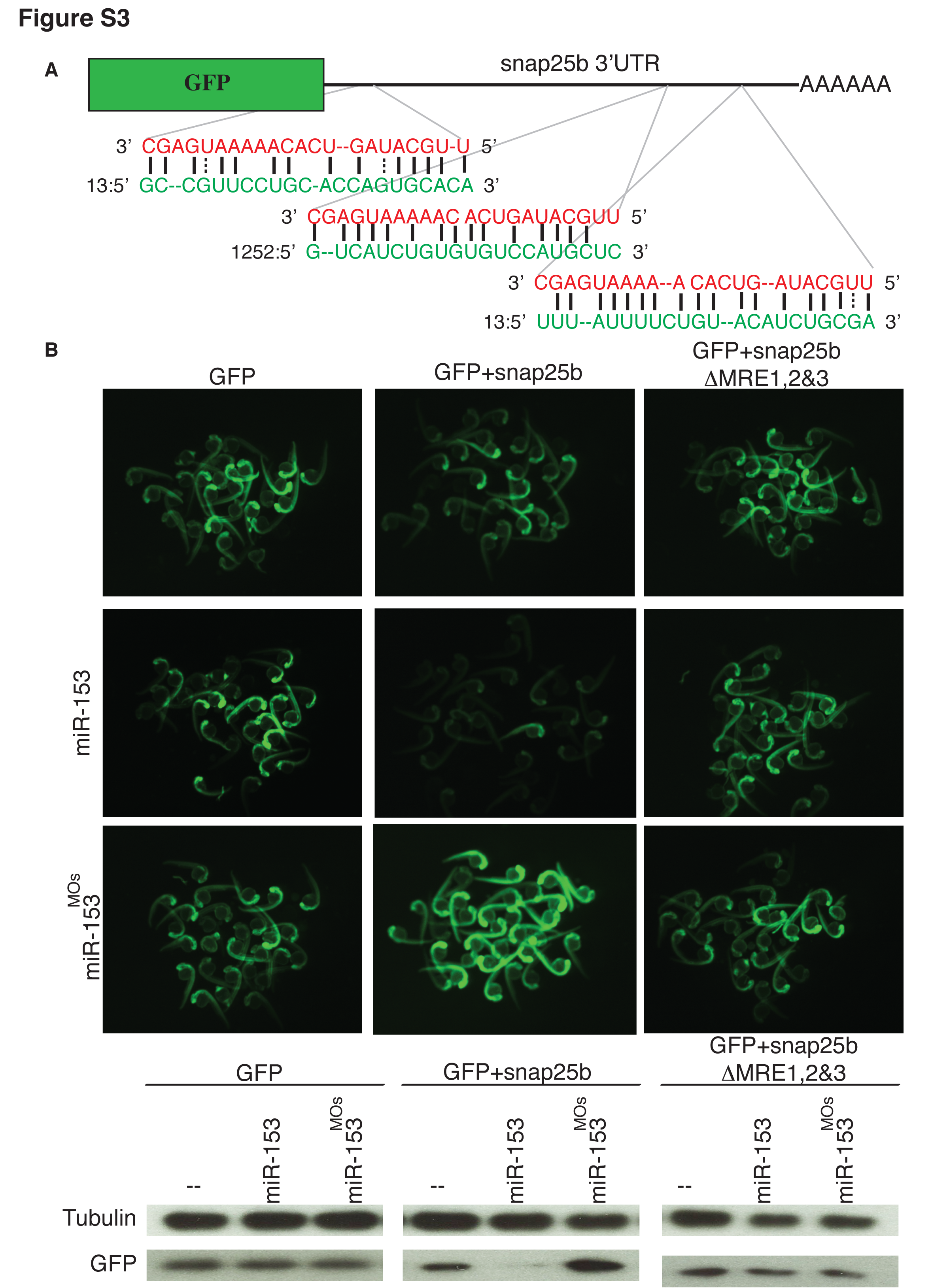

Supplement: Figure S3 — miR-153 targets snap-25b . (A) GFP reporter constructs were created by fusing the reading frame of GFP to the snap-25b 3′UTR. Three predicted miRNA recognition elements (MREs) were identified in the snap-25b 3′ UTR. The miR-153 sequence is indicated in red and the corresponding snap-25a UTR sequence is shown in green. (B) Single cell zebrafish embryos were injected with mRNAs derived from GFP reporters lacking a UTR (GFP), fused to the full length snap-25b UTR (GFP+snap-25b), or mutant version of the snap-25b UTR lacking all MREs (GFP+snap-25bΔMRE1, 2&3). Embryos were injected in the presence or absence of exogenous miR-153 or morpholinos against miR-153 (miR-153MO). Fluorescence levels were examined at 1 dpf. Clusters of embryos (∼30) are shown. (C)Lysates from ∼100 embryos were prepared from embryos treated as in B and GFP protein levels were determined by western blotting using antibodies against GFP or control antibodies against α-tubulin. (TIF) [file pone.0057080.s003.tif]

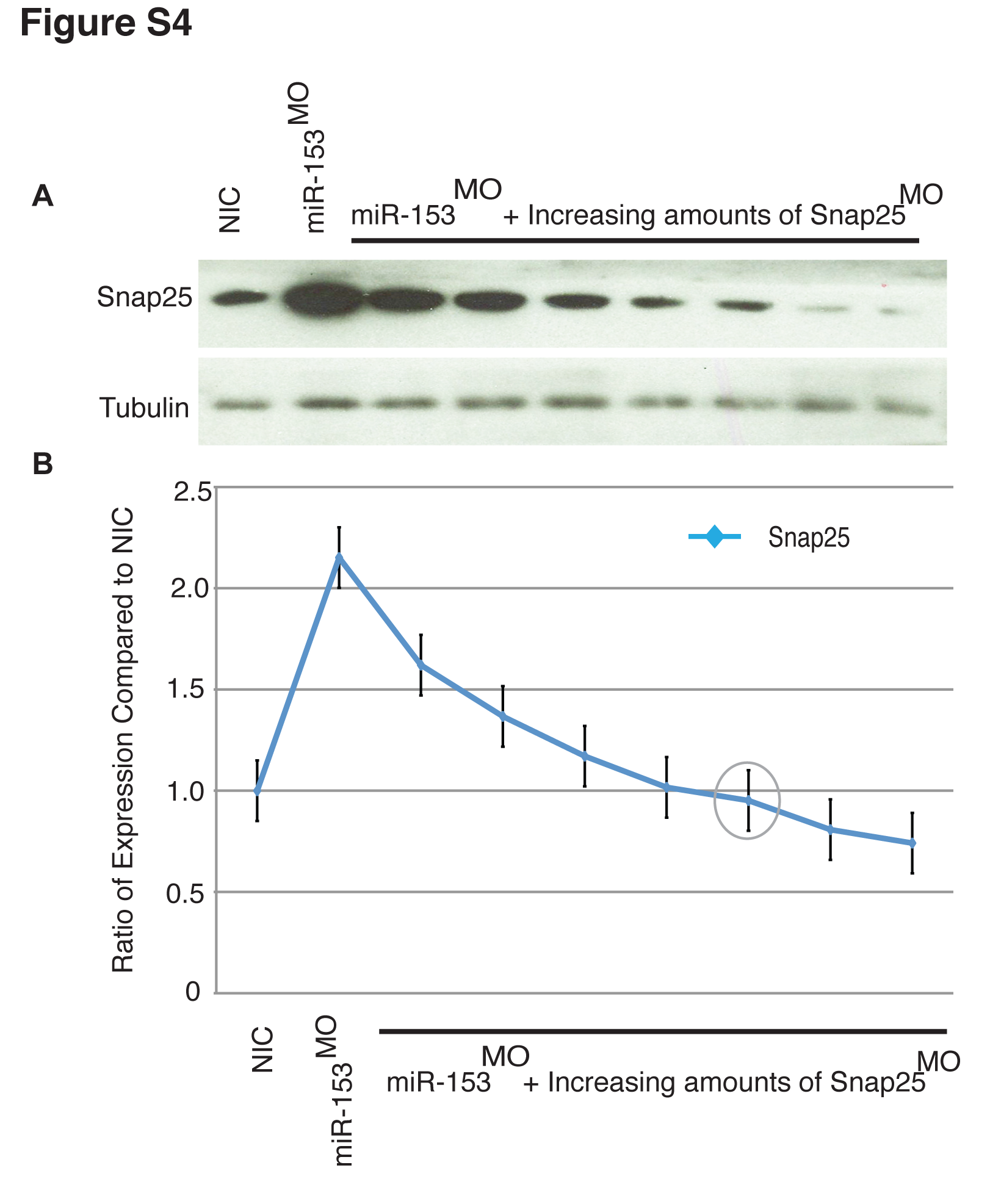

Supplement: Figure S4 — Dose-dependent rescue of miR-153 knockdown. (A) Single cell embryos were injected with a constant level of miR-153MO and increasing amounts (increments of 2 ng) of snap-25MOs. Embryo lysates from ∼60 embryos in each group were prepared and SNAP-25 protein levels determined by western blotting. (B) Quantitation of westerns (n = 3) from A. The grey circle represents the amount of snap- 25MO (10 ng) used in co-injection rescue experiments. (TIF) [file pone.0057080.s004.tif]

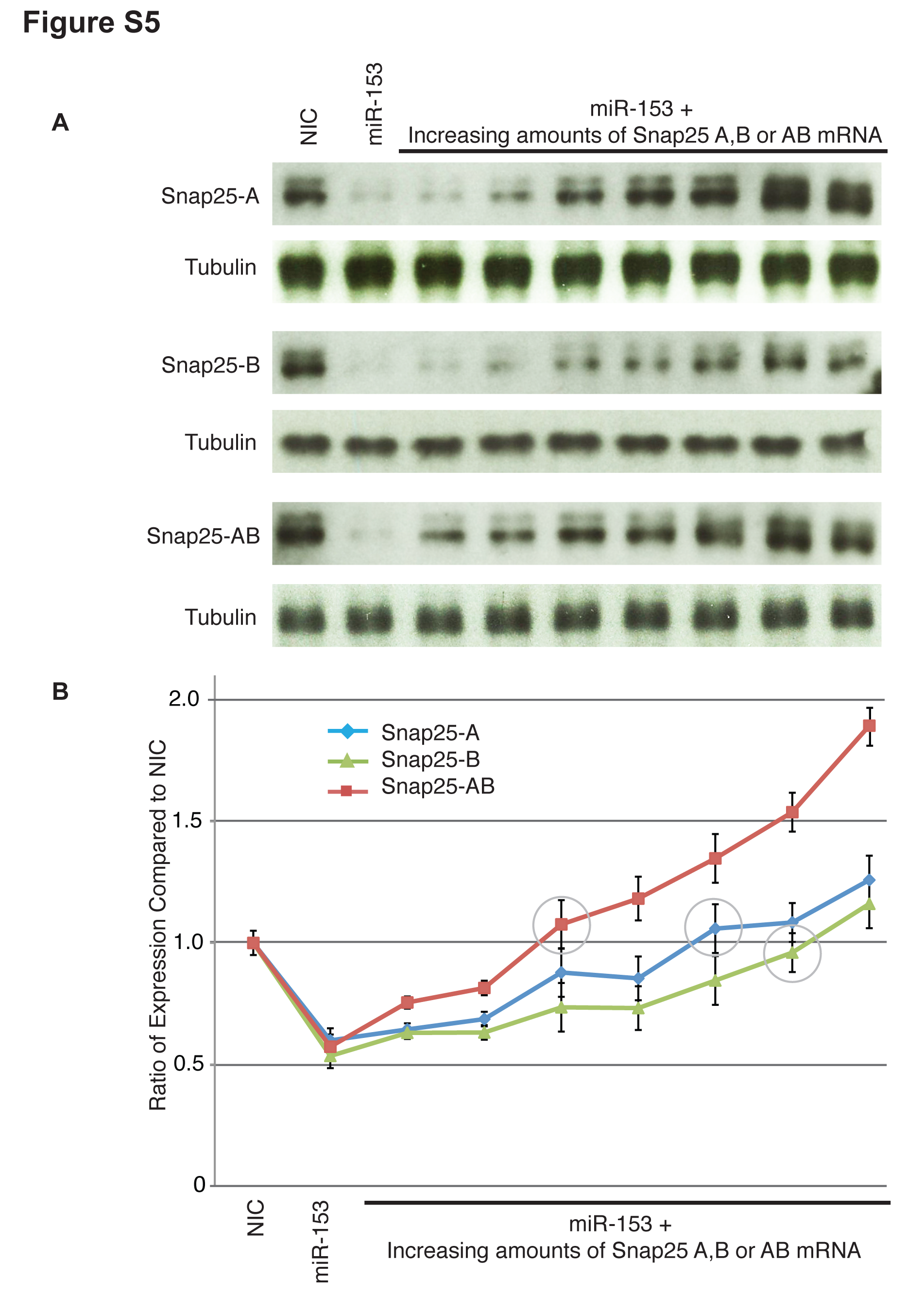

Supplement: Figure S5 — Dose-dependent rescue of miR-153 over-expression. (A) Single cell embryos were injected with a constant level of miR-153 and increasing amounts (increments of 50 pg) of snap-25a, snap-25b, or snap-25a&b mRNA. Embryo lysates from ∼60 embryos were prepared from embryos in each treatment group and SNAP-25 protein levels were determined by western blotting. (B) Quantitation of westerns (n = 3) from A. The grey circles represent the amounts used in co-injection rescue experiments (75 pg each of snap-25a and b, 250 pg of snap-25a, and 300 pg of snap-25b). (TIF) [file pone.0057080.s005.tif]

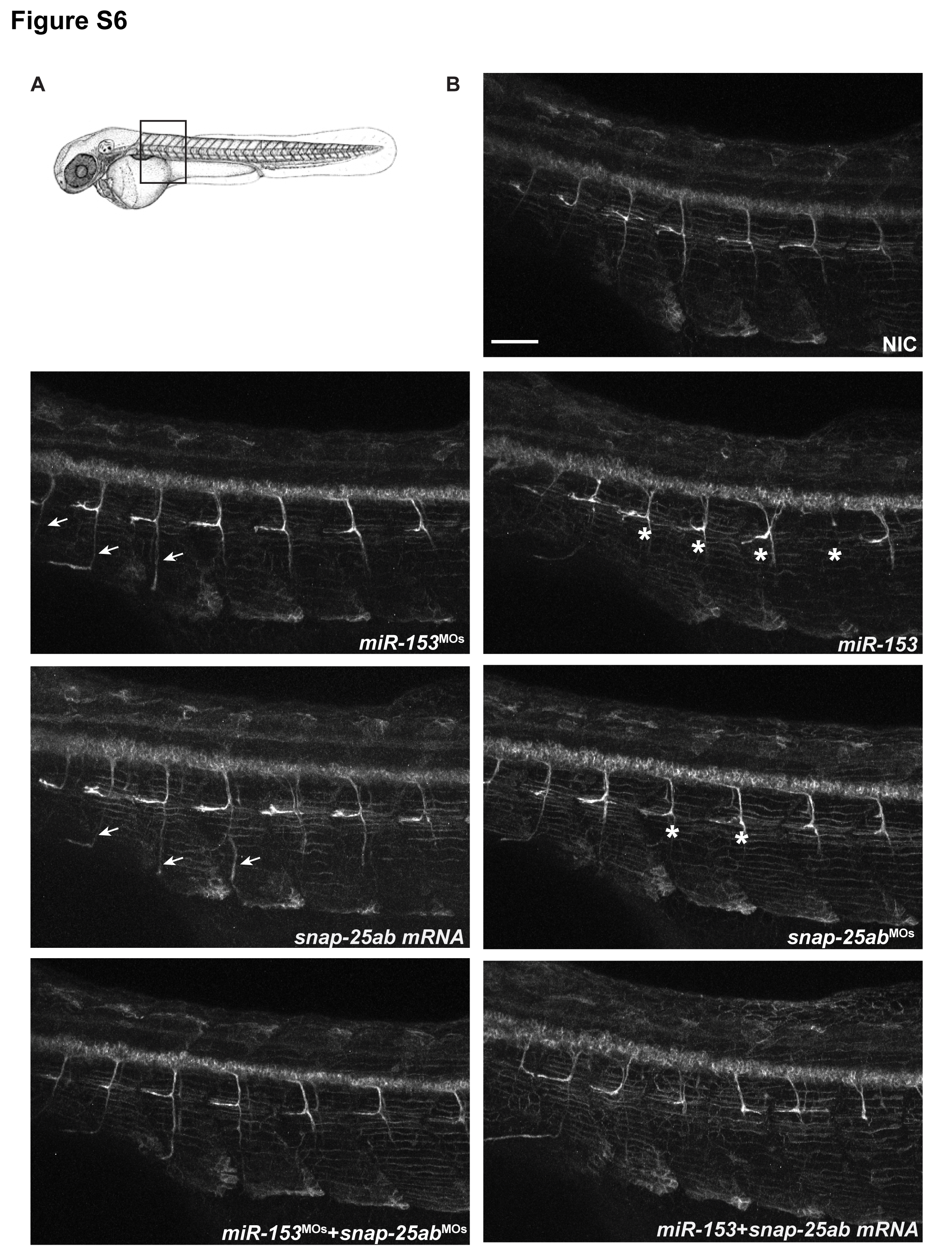

Supplement: Figure S6 — miR-153 regulates secondary motor neuron development. (A) Immunofluorescence was performed on whole mount zebrafish embryos at 55 hpf using Zn-8 antibodies to label secondary motor neurons. Confocal images were acquired from the same somites for all embryos, as indicated. (B) miR-153 knockdown (miR-153MO) and snap-25a,b overexpression significantly increased the growth of secondary motor neuron axons (arrows). Overexpression of miR-153 or knockdown of snap-25a,b (snap-25a,bMO) caused severe defects in axon development and architecture (asterisks). Scale bar: 40 µm. (TIF) [file pone.0057080.s006.tif]
